# Editorial–Special Issue on “Sensor Technology for Enhancing Training and Performance in Sport”

**DOI:** 10.3390/s23052847

**Published:** 2023-03-06

**Authors:** Pui Wah Kong

**Affiliations:** Physical Education and Sports Science Academic Group, National Institute of Education, Nanyang Technological University, Singapore 637616, Singapore; puiwah.kong@nie.edu.sg

## 1. Introduction

Sensor technology opens up exciting opportunities for sports. For example, advancements in motion tracking devices allow monitoring athletes’ movement patterns indoors and outdoors [1,2,3]. Wearable sensors offer insightful information on the demands of the sport during both training and competition [4]. Such data are important for coaches and athletes to optimize their training plan, minimize the risk of injuries, and improve sport performance. This Special Issue focuses on the innovative development and application of sensors for enhancing training and performance in sport. We received a total of 9 original and 1 review articles covering a variety of sports, including those on land [5,6,7,8,9,10,11,12,13] and in water [14].

## 2. Swimming

Providing performance feedback to swimmers is important for enhancing their training and competition but placing sensors in water can be technically challenging. A systematic review paper by Morais and colleagues [14] comprehensively summarized the currently available wearables that can provide real-time feedback in swimming. They reported that most systems are in-house built wearables with very few commercially available systems. The wearables were mostly placed on the lower back (44.4%) or the head (38.9%). Many studies assessed the accuracy, measurement errors, and/or consistency of the wearables and the majority reported the current swimming wearables as accurate. As the authors have pointed out that most researcher focused only on the front-crawl stroke, future work can expand the scope into other swimming strokes such as the breaststroke, backstroke, and butterfly stroke. We can also look forward to more commercially available wearables for swimming and other types of aquatic activities.

## 3. Global and Local Positioning Systems

Global Navigation Satellite System (GNSS) are wearables that allow continuous tracking of movement patterns of one or multiple players. Such technologies have been widely applied in sports such soccer, rugby, and field hockey. In this special issue, we published two articles concerning the applications of GNSS in sports. Chahal et al. [10] evaluated the inter-unit consistency and validity of multiple 10-Hz GNSS units in measuring straight-line sprint performance. Their participants worn 8 Catapult GNSS units at once and performed a maximum effort sprint. Poor inter-unit consistency for distance and speed measurements were found. For validity, most units recorded greater total distances and lower peak speeds than the reference values. Since variations exist among different units of the same GNSS system, the authors advised that if logistically possible, players should wear the same unit every time rather than swapping the units among teammates. This practice would allow a more meaningful comparison of the GNSS data of the player over a season. Another article by Bursais and colleagues [9] assessed and compared the ability of four accelerometry-based metrics and GNSS to predict the known distance completed using different movement constraints. In their study, participants were asked to walk around small and big circles of known distances. Acceleration data were collected via a tri-axial accelerometer at 100 Hz while positional data were sampled at 10 Hz using a triple GNSS unit. They reported promising regression model results, suggesting that both GNSS and accelerometry may be used to indicate the total distance completed while walking.

While GNSS are commonly used for outdoor sports, such systems do not work for indoor activities. As such, local positioning systems are developed to study match demands for indoor sports. In this special issue, one article by Guignard et al. [6] attempted to automatically define defensive organizations in professional handball with the use of local positioning systems. The research team first developed an automatic tool to detect and classify the defensive organization of the team. They then quantified the match demands per defensive organization. Using player data from a team in the Spanish League, the algorithm developed successfully quantified the physical demands of the game (distance stand, walk, jog, run, and sprint). Their results are promising, opening new opportunities for optimizing specific players’ roles as well as game strategies (e.g., defensive organization) at the team level.

## 4. Validity and Reliability

Different types of wearables sensors have been developed to support training for individuals participating in different types of sports. The validity and reliability of the wearables or smart devices may vary depending on the exercise intensity and the types of activities. With wrist-worn wearables becoming increasingly available, Cai and colleagues [7] investigated the reliability and validity of the Lexin Mio smart bracelet in physiological responses in 65 individuals with different physical activity levels exercising at different intensities. They found that the Lexin Mio smart bracelet showed good reliability and validity for heart rate measurement but low validity for the estimation of energy expenditure. In the sport of indoor cycling, Fiolo et al. [11] evaluated the validity and reliability of a tire pressure sensor cycling power meter against a gold standard. Twelve recreationally active participants completed 8 trials of 90-s cycling at different pedaling and gearing combinations while power outputs were measured. It is concluded that the tire pressure sensor can provide an accurate and reliable assessment of average power over a timeframe of 1-min duration or longer on an indoor roller trainer. The authors recommended this tool for practical use during general exercise or power with power outputs below 300 W and with less stringent error tolerance (±5%). Future research for outdoor use and under additional testing parameters, such as long-duration cycling, standing, sprinting, and inconsistent pedaling are recommended. Our last example is on running which is a very popular physical activity across many countries. Chow and colleague [13] compared the use of accelerometer and gyroscope in predicting running kinematics. Their team explored the use of convolutional neural network (CNN) to predict selected running kinematics (e.g., knee and hip angles) during level-ground running on a treadmill. The results reported that kinematics predictions in the sagittal plane were better for the knee joint than for the hip joint, and that predictions using the gyroscope as the regressor were better than those using the accelerometer.

## 5. Application of Wearables in Sports

After wearable sensors are developed and validated, it is of interest to see how they can be applied in real-life sport settings. This Special Issues included 3 articles demonstrating how different types of sensors are applied in field hockey [5], tennis [8], and running [13]. Cuadrado-Peñafiel et al. [5] compared the efficiency and effectiveness of two different configurations of within-season training load distribution in professional field hockey players over 6 weeks. They applied many types of equipment and sensors to monitor the players’ performance, for example, Global Positioning System (GPS), inertial measurement units (IMU), and Dynaspeed linear motorized system. The authors concluded that implementing strategies such as microdosed training load distribution can be an effective and efficient alternative for sprint training in team sports such as hockey. In tennis, Kramberger et al. [8] performed a case study to demonstrate how a wearable device can potentially contribute towards injury prevention in tennis. This study used the Armbeep wearable device to monitor the workload and recovery of a tennis player over 6 months. Using the data collected, the researchers instructed the coach and the player to adjust the daily workload with a goal to optimize the level of an athlete’s training load, increase the effectiveness of training, enable an individual approach, and reduce the possibility of overuse or injuries. This study is a real-life practical example of the use of modern technology in the supporting athletes’ training and competition over a sustained period of time.

Finally, one randomized controlled trial was conducted to examine the effectiveness of sensor-based gait retraining in lowering risk factors and symptoms of knee osteoarthritis (OA) [12]. This study randomized 71 participants with early medial knee OA in to either walking exercise or gait retraining groups. During the gait retraining intervention, real-time visual feedback was provided based on IMU measurements while participants were walking on a treadmill. The authors concluded that a six-week sensor-based gait retraining, compared with walking exercise, was an effective intervention to lower medial knee loading, relieve knee pain, and improve symptoms for patients with early medial knee OA.

## 6. Summary

I am delighted to see that the research articles in this Special Issue have generated new knowledge to the field of sensor technology for sports, identified research gaps for future advancement, and opened new opportunities for innovation and collaboration. As a final remark, I would like to thank all the authors for their contributions to this Special Issue. My thanks also go to the reviewers for their insightful comments and helpful feedback for the authors.

## Data Availability

Not applicable.
